# Development and Validation of a Tool for Evaluating Self-regulated and Self-directed Aptitudes of Learning (SELF-ReDiAL)

**DOI:** 10.1007/s40670-025-02454-0

**Published:** 2025-07-04

**Authors:** Arash Arianpoor, Silas C. R. Taylor, Cherie Lucas, Craig S. Webster, Marcus A. Henning, Ernesta Sofija, Matthew J. Boyd, Theresa L. Charrois, Jamie Kellar, Jason Perepelkin, Lorraine Smith, Revathy Mani, Efi Mantzourani, Catherin Marley, Boaz Shulruf, Pin-Hsiang Huang

**Affiliations:** 1 Office of Medical Education, Faculty of Medicine and Health, The University of New South Wales, Sydney, NSW Australia; 2 School of Biomedical Sciences, Faculty of Medicine and Health, The University of New South Wales, Sydney, NSW Australia; 3 School of Population Health, Faculty of Medicine and Health, The University of New South Wales, Sydney, NSW Australia; 4 Centre for Medical and Health Sciences Education, School of Medicine, University of Auckland, Auckland, New Zealand; 5 Department of Anaesthesiology, Faculty of Medical and Health Sciences, University of Auckland, Auckland, New Zealand; 6 School of Medicine and Dentistry, Griffith University, Gold Coast, QLD Australia; 7 Division of Pharmacy Practice and Policy, School of Pharmacy, University of Nottingham, Nottingham, UK; 8 Faculty of Pharmaceutical Sciences, The University of British Columbia, Vancouver, BC Canada; 9 Leslie Dan Faculty of Pharmacy, University of Toronto, Toronto, Canada; 10 College of Pharmacy & Nutrition, University of Saskatchewan, Saskatoon, SK Canada; 11 Sydney Pharmacy School, Faculty of Medicine and Health, The University of Sydney, Sydney, NSW Australia; 12 School of Optometry and Vision Science, Faculty of Medicine and Health, The University of New South Wales, Sydney, NSW Australia; 13 School of Pharmacy and Pharmaceutical Sciences, Cardiff University, Cardiff, Wales UK; 14 Faculty of Medicine and Health, The University of New South Wales, Sydney, NSW Australia; 15 Department of Medical Humanities and Medical Education, College of Medicine, National Yang Ming Chiao Tung University, Taipei, Taiwan

**Keywords:** Lifelong learning, Self-directed learning, Self-regulated learning, Health professional education, Medical education, Scale development

## Abstract

**Introduction:**

Self-regulated learning (SRL) and self-directed learning (SDL) are widely studied in education, but debates about their relationship have hindered effective measurement in practice. The recently introduced SELF-ReDiAL framework (self-regulated and self-directed aptitudes of learning) addresses this by framing these as adaptable learning aptitudes, integrating SRL features and insights into SDL. Using this framework, we developed and validated a new tool to assess SELF-ReDiAL—particularly valuable for health students and professionals requiring lifelong learning—bridging educational theory and practice.

**Methods:**

Guided by the SELF-ReDiAL framework, a 30-item questionnaire was developed and administered to students in health-related disciplines across Australia, New Zealand, the UK, and Canada. Exploratory and confirmatory factor analyses (EFA and CFA) assessed the scale’s content and construct validity.

**Results:**

Overall, 315 responses were analysed (mean age: 23.20 ± 6.73 years, range: 17–58), including 241 women, 70 men, and 4 individuals using other gender terms. Following EFA, 20 items were retained, yielding a four-factor model: ‘Inquisitiveness’ (31.17% variance explained), ‘Accomplishment’ (4.46% variance explained), ‘Implementation’ (4.11% variance explained), and ‘Independence’ (2.54% variance explained). CFA confirmed model fit (*χ*^2^ = 374.334, *df* = 164, *p* < 0.01, *χ*^2^/*df* = 2.283; CFI: 0.91, TLI: 0.896, RMSEA: 0.064, SRMR: 0.0523). Both Cronbach’s alpha and composite reliability closely met the threshold for all factors.

**Discussion:**

The SELF-ReDiAL model offers a comprehensive perspective on learners’ ability to take ownership of their learning when addressing gaps in professional knowledge. In health education, assessing SELF-ReDiAL helps identify influencing factors and informs strategies to enhance these aptitudes, prompting lifelong learning and ensuring high-quality patient care.

**Supplementary Information:**

The online version contains supplementary material available at 10.1007/s40670-025-02454-0.

## Introduction

Modern health education places significant emphasis on developing health professionals as lifelong learners [1]. This focus has garnered increasing attention in recent years, driven by major transitions to online, remote, and hybrid education prompted by events such as the COVID-19 pandemic and advancements in generative AI, alongside the challenges of a rapidly changing and unpredictable modern society [2, 3].

Lifelong learning is closely linked to self-regulated learning (SRL) and self-directed learning (SDL). Therefore, when the abilities for lifelong learning are required to be evaluated in learners, this can, at least in part, be achieved through an evaluation of their preparedness for both SRL and SDL [1]. Extensive research over the past few decades has examined both SRL and SDL [4–9], and studies across different fields, including health professions, have explored how levels of and readiness for SRL and SDL relate to learning outcomes [10, 11].

SRL has traditionally been defined as ‘…the self-directive process by which learners transform their mental abilities into academic skills…’ through ‘self-generated thoughts, feelings, and behaviours that are oriented to attaining goals’ [5]. According to Zimmerman, the cyclical process of SRL begins with the *forethought phase*, emphasising two subprocesses: (i) task analysis, which involves goal setting and strategic planning; and (ii) self-motivation, which stems from the learners’ beliefs about their capabilities, expectations, interests, and values [5]. This phase is followed by a *performance phase*, entailing self-control and self-observation. The final phase requires *self-reflection* and involves self-judgement. In this phase learners reflect on their educational processes through self-evaluation and causal attributions (i.e. beliefs about the cause of their errors or successes). A key component of this phase is self-reaction, which includes elements of self-satisfaction and adaptive/defensive responses [5].

SDL involves more self-explanatory steps: ‘…a process in which individuals take the initiative, with or without the help of others, in diagnosing their learning needs, formulating learning goals, identifying human and material resources for learning, choosing and implementing appropriate learning strategies, and evaluating learning outcomes’ (p.18) [7]. In a formal setting facilitated by an educator, SDL involves collaborative processes between the educator and learner. This includes jointly planning the learning process through participative decision-making, diagnosing needs via mutual assessment, setting goals through negotiation, and evaluating outcomes through the shared assessment of self-collected evidence [7]. However, it is important to note that SDL is not confined to the boundaries of the classroom, but extends throughout life, driven by personal values and aspirations [12]. Formal education is just one part of the lifelong learning process, with SDL contributing, at least partially, to the learner’s development throughout life [12, 13].

Current literature on SRL and SDL reveals foundational similarities between these two theories, as both are composed of ‘internal monitoring’ (covert aspect) and ‘external management’ (overt aspect), both require active involvement of the learners, and both emphasise the learners’ agency in taking responsibility and control [4, 14]. However, differences also exist between SRL and SDL. Cosnefroy and Carré outline these distinctions across the following three key dimensions [14]. First, in terms of their field of reference, SRL stems from educational psychology, while SDL arises from adult education. Second, their traditional target populations differ: SRL focuses on children and adolescents, while SDL primarily addresses learning in adults. Finally, while SRL is more concerned with formal academic situations, SDL mainly involves learning projects outside the classroom, where learners have the agency to define their needs and tasks.

Over the past decades, researchers have developed various tools to measure SRL and SDL in learners [15–17], some of which focus on health students and professionals [9, 18–20]. However, there is no consensus on which tools are most appropriate for measuring SRL and SDL. This lack of agreement, coupled with the long-standing debate about the associations between SRL and SDL and their differences and similarities [4, 14, 21], has led to a call for translating these two entangled theories into educational practice by developing a comprehensive framework that represents both [4, 12, 14].

To address this call, drawing on prevalent descriptions of SRL [5, 8], SDL [7], and the traditional definition of aptitude [22], a comprehensive framework was recently conceptualised by our team to view these as flexible and adaptable learning aptitudes, labelled as *self-regulated and self-directed aptitudes of learning* (SELF-ReDiAL, or SR for brevity) [23]. According to this framework, learners with SR are capable of recognising a learning need when a challenge presents; they define goals to meet that need and choose appropriate learning strategies to reach their goals. These learners are aware of their own cognitive processes, aligning them toward their learning goals and constantly monitoring the entire learning process [23]. More importantly, learners with high levels of SR are motivated to initiate their own learning when they recognise the need for it.

The SR framework views SRL and SDL practices in learners generally, regardless of their application—in a single learning task, within the classroom, or in informal settings—as aptitudes for learning. It represents the initial or general status of learners, which impacts their development. Rather than being merely a learning skill, SR reflects the preparedness of learners to face any learning situation, whether instructed in a classroom or self-directed during practice [23]. This is particularly crucial for health professionals, whose education is uniquely characterised by dynamic clinical learning environments, evolving healthcare demands, and the continuous emergence of new diseases and societal health requirements. These features necessitate professionals with a strong aptitude for lifelong learning [1], embodied in higher levels of SR [23].

The SR framework offers insights into SDL while also incorporating the important features of SRL. Given that self-regulation is a prerequisite for a learner to become capable of SDL—meaning that SDL encompasses SRL, while the opposite might not necessarily be true [14, 21]—this perspective is essential. Recognising the specific importance of SR for health professionals and the lack of existing tools that simultaneously measure both SRL and SDL, we aimed to develop and validate a new tool by adopting the aforementioned comprehensive perspective of SR [23], enabling a more holistic evaluation of these learning aptitudes.

## Methods

### Scale Construct

Referring to the framework presented for SR [23] and through consensus among the authors, items aligning with this conceptual framework were selected from three existing self-report scales: the motivated strategies for learning questionnaire [15] and SRL perception scale [20] for SRL, and the SDL readiness scale [9] for SDL. These selected items underwent refinement to establish clarity and consistency, and supplementary author-developed items were then incorporated to fill conceptual gaps, ensuring a thorough representation of the components of SR. In the subsequent phase, a pilot study was conducted to further refine the items for clarity. In this pilot, a preliminary version of the questionnaire was distributed to a small group (*n* = 4) of medical students in their clinical years for feedback. Based on their feedback, one item was removed due to perceived similarity to another, and the wording of six items was adjusted to enhance clarity. The resultant questionnaire consisted of 30 items (see Online Resource 1, Table S1). The items were further refined to ensure positive wording and were anchored to a six-option frequency Likert-like scale ranging from ‘never’ to ‘always’. The decision to use a frequency scale with positively worded items was grounded on the superiority of such scales over agreement scales in mitigating susceptibility to acquiescence bias [24]. Additionally, the six-option scale serves two purposes: (i) it avoids a mid-point response, encouraging the participants to choose an option rather than opting for a neutral position, and (ii) it allows for discrimination between responses, enabling the use of conventional parametric statistics [24].

### Sample

To ensure robust results across different contexts in health professions, we aimed to include students who, at the time of the survey distribution, were enrolled in health-related disciplines such as Medicine, Pharmacy, Exercise Physiology, Optometry, and other health disciplines at universities in Australia, New Zealand, the UK, and Canada. The inclusion of universities in these regions was based on curriculum similarities and a shared language, and all universities offering health-related programs were eligible for inclusion.

The online survey, including the SR Scale items and background information (e.g. sex, age, and nationality/culture), was generated using Qualtrics™ (Provo, UT, USA). The link was distributed through program authorities—who were not teaching any students involved in the study, thereby minimising potential bias from power dynamics—via broadcast emails, announcements, website and newsletter advertisements, and social media. The distribution occurred in two stages: the first was timed by the program authorities, based on key timepoints in the term to avoid adding stress during critical periods (e.g. assessments), and the second was a reminder sent 2 weeks after the initial announcement.

To determine the required sample size, the generally accepted 10:1 ratio of observations to variables was applied [25], suggesting a minimum of 300 responses to validate the 30-item scale.

### Data Management and Statistical Analysis

All responses were extracted from Qualtrics™ to Microsoft Excel®. Cases were screened for missing data and unengaged responses. A standard deviation (SD) threshold of ≤ 0.50 was applied as a rule of thumb to identify cases with unengaged responses and determine their exclusion [26].

Exploratory factor analysis (EFA) was performed using maximum likelihood factor analysis with oblimin rotation [27–29]. Furthermore, to determine the appropriate number of factors to extract, eigenvalues were calculated, and a scree plot was generated. As per the guidelines provided by Hair et al. [25], factor loadings ≥ 0.4 were considered significant for retaining items in this study, while items with cross-loadings on two or more factors (Δ factor loadings ≥ 0.2) were carefully examined [30]. Reliability for each extracted factor was then assessed using Cronbach’s alpha. In accordance with general principles for naming extracted factors [27, 28], each factor was named based on the common conceptual themes among the items loaded on that factor.

Subsequently, to complement the EFA, confirmatory factor analysis (CFA) was performed [31] on the same sample [32], incorporating additional fit measures including the chi-square (*χ*^2^), degrees of freedom (*df*), comparative fit index (CFI), Tucker-Lewis index (TLI), root mean square error of approximation (RMSEA), and standardised root mean square residual (SRMR). To address the potential multivariate non-normality in real-world data, and for assessing the (in)stability of statistical models across a range of population compositions, bootstrapping was performed with 1000 bootstrap samples, providing 95% bias-corrected confidence intervals for parameter estimates [31, 33].

To evaluate model fit across different groups, multiple groups invariance analyses [31] were performed based on gender (female vs. male, excluding other genders due to insufficient responses) and course of study (Medicine, Pharmacy, and other health-related disciplines).

All analyses were conducted using IBM SPSS Statistics (Version. 29. Armonk, NY: IBM Corp. 2023) and AMOS (Version. 29. Armonk, NY: IBM Corp. 2023).

### Ethics

The study received approval from the ethics committee of UNSW (reference numbers: HC230102 and iRECS5767). This approval was recognised by the ethics committees of participating universities, and reciprocal approvals were gained where required.

All responses were submitted anonymously, and no identifiable information, including metadata, were recorded. Participants provided consent for participation through an online Participant Information Statement and Consent Form, using an opt-in procedure.

## Results

### Participants

Overall, thirteen universities agreed to participate in this study. A total of 436 responses were collected. Of these, 87 incomplete responses and 17 responses from non-health students were excluded. Additionally, 7 responses were excluded as they were deemed unengaged (SD ≤ 0.5; see ‘Data Management and Statistical Analysis’ section of ‘Methods’). Finally, to mitigate missing values for EFA and CFA, all responses with missing values for SR Scale items (*n* = 10) were omitted, leaving 315 responses for inclusion in the analysis.

The mean age of participants was 23.20 ± 6.73 years (range: 17–58 years). Among the respondents, 241 identified as women/females, 70 as men/males, and 2 as non-binary. Additionally, one respondent indicated using another gender term, and one did not answer the question. In terms of year of study, 103 students were in their first year, 69 in their second year, 69 in their third year, 48 in their fourth year, 15 in their fifth year, 4 in their sixth year, and 7 did not specify their year of study. Of the 315 observations included in the analysis, 145 were from students enrolled in Australian universities (The University of New South Wales, Griffith University, The University of Tasmania, The University of Sydney, and the Joint Medical Program (JMP) at University of Newcastle and University of New England), 83 from universities in New Zealand (The University of Auckland), 61 from Canadian universities (The University of Saskatchewan, University of Toronto, The University of Alberta, and The University of British Columbia), and 26 from universities in the UK (The University of Nottingham and The University of East Anglia). Distribution of courses of studies is provided in Online Resource 1 (Table S2).

### Exploratory Factor Analysis

EFA was conducted on the full sample (*n* = 315, with no missing values for SR Scale items) using a threshold of 1.2 for eigenvalues and 250 iterations. The Kaiser–Meyer–Olkin (KMO) measure of sampling adequacy (0.922) and Bartlett’s test of sphericity (*χ*^2^ = 3703.427, *df* = 406, *p* < 0.001) confirmed the suitability of data for factor analysis. After reviewing the items from a theoretical perspective, along with factor loadings, cross-loadings, and reliability values, and following discussions among the authors, 10 of the initial 30 items were removed (see Online Resource 1, Table S3). Notably, item SR12 (‘I can learn anything relevant to my needs in my field of study’) was excluded due to content similarity with SR8 (‘I can learn anything relevant to my needs’) and was not included in the EFA, while nine items were removed due to either loadings < 0.4 or cross-loading on more than one factor.

According to the EFA results, a four-factor model emerged with 20 retained items. The first factor comprised items related to ‘Inquisitiveness’ (eight items, explaining 31.17% of the variance), the second to ‘Accomplishment’ (five items, explaining 4.46% of the variance), the third to ‘Implementation’ (four items, explaining 4.11% of the variance), and the fourth to ‘Independence’ (three items, explaining 2.54% of the variance). Factor loadings, based on the pattern matrix, for included items and factor groupings are presented in.

Table 1. Cronbach’s alpha was 0.860 for ‘Inquisitiveness’ and 0.835 for ‘Accomplishment’, both exceeding the accepted 0.70 threshold, and it was 0.693 for ‘Implementation’ and 0.683 for ‘Independence’, both just below the recommended threshold [25].

**Table 1:** Factor loadings of SELF-ReDiAL scale items from exploratory factor analysis

Code	Item	Factor loadings^a^			
		Inquisitiveness	Accomplishment	Implementation	Independence
SR5	I learn to satisfy my curiosity	**0.865**	− 0.002	− 0.147	− 0.062
SR1	I search for possibilities to learn new things	**0.619**	0.116	0.077	0.147
SR6	I enjoy learning new things	**0.593**	− 0.105	− 0.080	0.142
SR7	I seek to learn beyond the stated requirement	**0.562**	0.012	0.039	0.170
SR23	I deliberately integrate new knowledge with my existing knowledge	**0.534**	− 0.164	0.012	0.044
SR3	I learn to improve myself	**0.511**	− 0.108	0.187	0.146
SR14	I welcome challenges in learning	**0.507**	− 0.092	0.126	0.152
SR4	My reason for learning is to gain personal benefit	**0.450**	0.050	0.107	− 0.037
SR28	I meet my learning needs fully	0.153	** − 0.733**	0.057	− 0.035
SR27	I meet my learning needs on schedule	− 0.002	** − 0.701**	0.006	0.032
SR26	I meet my learning objectives	0.102	** − 0.596**	0.001	0.218
SR9	I am aware of my learning capabilities	0.135	** − 0.498**	− 0.091	0.158
SR25	I complete my learning despite challenges	0.035	** − 0.484**	0.108	0.245
SR30	I evaluate my learning	0.119	− 0.070	**0.647**	− 0.017
SR18	I set my own learning objectives	0.113	0.103	**0.607**	0.024
SR19	I plan my learning in advance	− 0.053	− 0.384	**0.455**	− 0.041
SR17	To start learning, I organise relevant available learning materials	− 0.072	− 0.108	**0.455**	0.128
SR13	I learn independently to other people	− 0.114	− 0.126	0.019	**0.629**
SR21	I attempt to independently solve learning challenges	0.134	− 0.007	0.026	**0.585**
SR10	I can find resources by myself	0.126	− 0.180	− 0.110	**0.530**

^a^Factor loadings are based on the pattern matrix from exploratory factor analysis using maximum likelihood extraction and oblimin rotation with Kaiser normalisation. Factor loadings ≥ 0.4 were considered significant for retaining items (marked in bold). Only retained items are shown

*SELF-ReDiAL* self-regulated and self-directed aptitudes of learning

### Confirmatory Factor Analysis

**Fig. 1:**
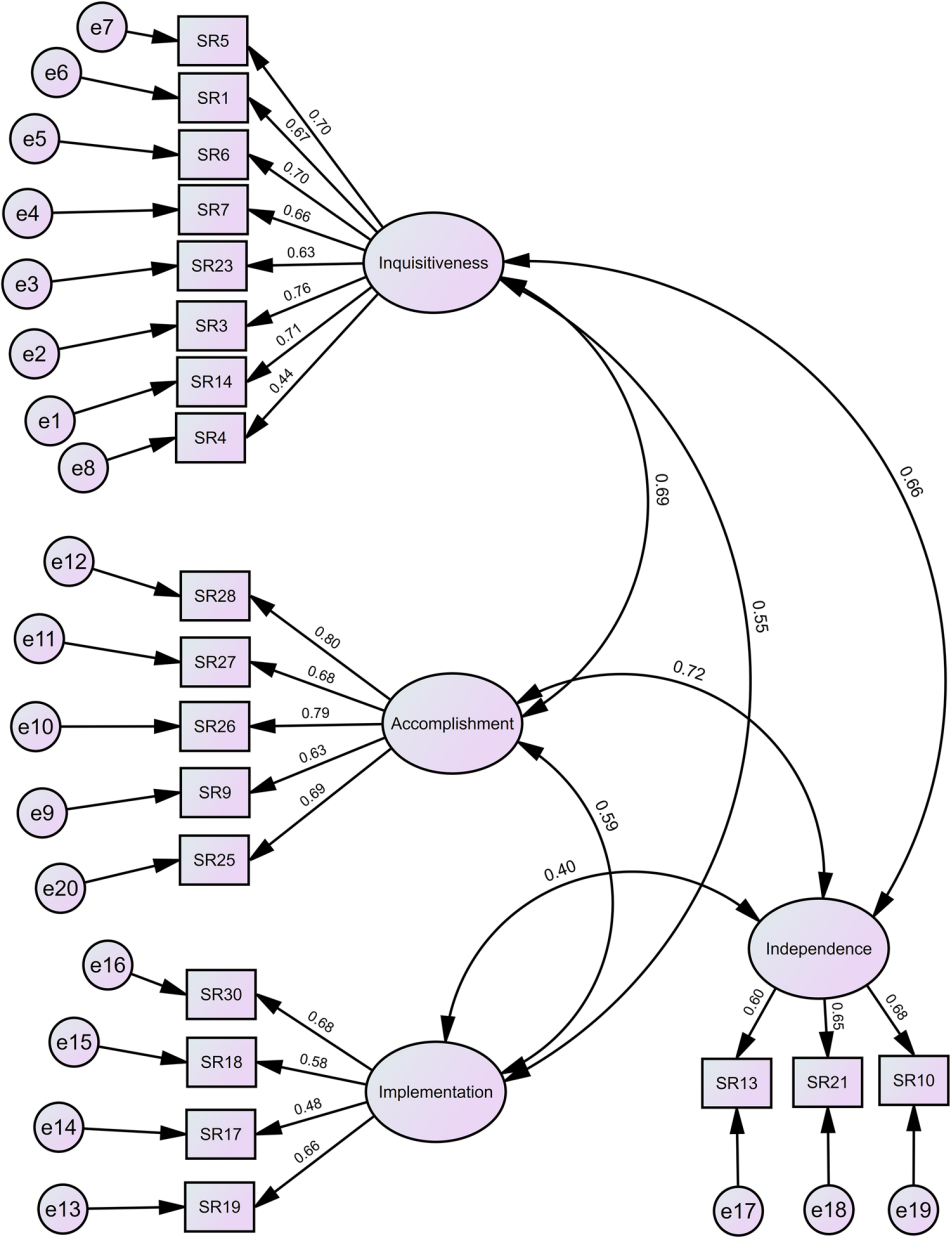
Structured model for self-regulated and self-directed aptitudes of learning (SELF-ReDiAL). This figure presents the final model based on confirmatory factor analysis (CFA), which itself is a refinement of the original model constructed through exploratory factor analysis (EFA). Ovals represent the latent variables, and rectangles represent the observed variables (i.e. items on scale; refer to Table 1). Single-headed arrows indicate standardised factor loadings (i.e. regression paths). Measurement errors are represented by circles (e1 to e19). The double-head arrow shows the correlation between the factors

To further assess the model fit, CFA was conducted on the same sample used in the EFA (*n* = 315) (Fig. 1). The results indicated a good fit based on the chi-square test (*χ*^2^ = 374.334, *df* = 164, *p* < 0.01, with *χ*^2^/*df* = 2.283 meeting the accepted threshold of < 3), CFI (0.91, meeting the accepted threshold of > 0.9), and TLI (0.896, just meeting the threshold of > 0.9). Additionally, the values for RMSEA (0.064 [90% CI: 0.055–0.072]; accepted threshold ≤ 0.08) and SRMR (0.0523; accepted threshold < 0.08) were within the acceptable range [25, 34]. Bootstrapping analysis demonstrated the reliability and stability of the CFA model, with low biases, small standard errors, and confidence intervals that consistently excluded zero [31] (see Online Resource 1, Table S4). Moreover, composite reliability (CR) values met the accepted 0.70 threshold for all factors. ‘Inquisitiveness’ and ‘Accomplishment’ demonstrated strong CRs of 0.87 and 0.85, respectively, while ‘Implementation’ and ‘Independence’ closely approached the threshold with CRs of 0.70 and 0.69, respectively, indicating robust internal consistency and factor reliability. Moderate correlations were observed between the factors (Fig. 1).

### Multiple Groups Invariance Analyses

In terms of configural invariance, the model was well-fitting across genders (241 women/female and 70 men/male; *χ*^2^ = 549.336, *df* = 328, *χ*^2^/*df* = 1.675; CFI = 0.906; TLI = 0.891; RMSEA = 0.047) and courses of study (Medicine: 108, Pharmacy: 92, and other health-related disciplines: 115; *χ*^2^ = 842.386, *df* = 492, *χ*^2^/*df* = 1.712; CFI = 0.859; TLI = 0.837; RMSEA = 0.048). Additionally, in terms of measurement invariance, the scale demonstrated good metric invariance across these groups (gender: Δ*χ*^2^ = 24.982, Δ*df* = 16, *p* = 0.070; course: Δ*χ*^2^ = 21.670, Δ*df* = 16, *p* = 0.154), suggesting consistent measurement of construct across these groups.

## Discussion

In this study, we aimed, for the first time, to develop a tool that taps into learners’ readiness for both SRL and SDL through the comprehensive lens of the SR framework. SR primarily focuses on learners’ willingness and ability to recognise learning needs at any time, their awareness and confidence in addressing those needs, and their capacity to set goals based on those needs and take action to meet them [23].

The validation study of the proposed scale identified four factors related to SR in health professions students: ‘Inquisitiveness’, ‘Accomplishment’, ‘Implementation’, and ‘Independence’, all of which align with the core aspects of SR.

### Inquisitiveness

According to our main analysis, ‘Inquisitiveness’ explained the largest proportion of variance (31.17%) and was shown to be a reliable indicator of SR (Cronbach’s alpha: 0.860). This aligns with the conceptualisation of *inquisitiveness* as an intellectual virtue—with the common goal of improving one’s beliefs, knowledge, and understanding—while being uniquely characterised by a tendency to question [35]. An inquisitive individual is ‘characteristically motivated to engage sincerely in good questioning’ (p. 43) [35]. This definition closely aligns with that of a learner with high levels of SR, who is considered to be motivated by a desire to learn [23].

Inquisitiveness, in the context of learning, manifests as a *purposeful curiosity*, fostering reflection, critical thinking, and a continuous pursuit of knowledge [36]. To better contextualise this, curiosity—a sister concept to *inquisitiveness*—is defined as the ‘… desire for knowledge or information in response to experiencing or seeking out collative variables which is accompanied by positive emotions, increased arousal, or exploratory behaviour’ (p. 37) [37]. In this definition, collative variables refer to factors such as novelty, complexity, ambiguity, challenge, disequilibrium, and uncertainty [37]—many of which align closely with the items loaded on ‘Inquisitiveness’.

A closer examination of these items highlights their relevance to various aspects of curiosity. For instance, item SR 1 (‘I search for possibilities to learn new things’) and item SR 6 (‘I enjoy learning new things’) reflect ‘novelty’ as one of the aspects of curiosity, while item SR 14 (‘I welcome challenges in learning’) represents ‘challenge’ as a trigger for information-seeking. Furthermore, higher levels of curiosity are associated with greater intrinsic motivation to explore beyond merely fulfilling assessment requirements or meeting outlined expectations [38], a concept well captured by item SR 7 (‘I seek to learn beyond the stated requirement’).

Inclusion of items SR3 (‘I learn to improve myself’), SR4 (‘My reason for learning is to gain personal benefit’), and SR5 (‘I learn to satisfy my curiosity’) under this factor aligns closely with the interest-type (I-type) curiosity. I-type curiosity arises when individuals perceive learning something new as enjoyable or intellectually stimulating [39]. In contrast, D-type curiosity (deprivation-type) arises from recognising a gap in understanding that creates a sense of discomfort until resolved with new information [39]. While items SR3 and SR4 primarily reflect the I-type curiosity, they also hint at a sense of disequilibrium (i.e. a perceived gap). Specifically, inquisitive individuals may identify areas in themselves that need improvement (SR3) and see personal benefit in addressing this disequilibrium (SR4). Thus, both forms of curiosity influence how individuals approach new information, the SRL strategies they employ, and the way they set SDL goals [39].

According to the definition of SR, learners with high levels of SR will engage in SDL activities when they identify a learning gap [23], seemingly aligning more with D-type curiosity. However, considering the items loaded on ‘Inquisitiveness’, we propose that learners with high SR levels are not only capable of addressing their learning needs but also demonstrate an intrinsic desire to learn for its own sake. This disposition, although not always actively expressed, remains an enduring aptitude, reinforcing their identity as lifelong learners.

### Accomplishment

The term *accomplishment* is defined as ‘the action or fact of accomplishing something; fulfilment, completion; achievement, success’ [40]. In formal education, *accomplishment* of articulated learning goals is generally equated with *student achievement* [41]. However, in informal learning environments, which are central to lifelong learning, the determination of *accomplishment* largely rests with the learner, who assesses the extent to which they have achieved their learning goals [42]. Given this, in the context of SR, *accomplishment* will be defined as the success of learners in meeting their learning needs. This is well captured by most of the items loaded on ‘Accomplishment’ (SR25: ‘I complete my learning despite challenges’; SR26: ‘I meet my learning objectives’; SR27: ‘I meet my learning needs on schedule’; and SR28: ‘I meet my learning needs fully’).

From another standpoint, items loaded on ‘Accomplishment’ reflect learners’ confidence in themselves for meeting their needs—interpretable as *self-efficacy*—as well as awareness of their own capacity to do so, which aligns more closely with *self-concept* [43]*.* This is best represented by item SR9 (‘I am aware of my learning capabilities’). Traditionally, self-efficacy has been incorporated into SRL theories as an integral component of the learning cycle [5, 8]. Higher self-efficacy in learners is associated with a greater willingness to initiate challenging learning tasks, increased effort and persistence in implementing those tasks, and lower levels of anxiety [43]. Given this theoretical perspective, it is unsurprising that ‘Accomplishment’ emerged as the second major factor explaining SR aptitudes in health professions students. Consequently, learners who perceive higher levels of *accomplishment* are expected to demonstrate higher levels of SR.

Notably, the results of CFA showed a large covariance (0.69, Fig. 1) between ‘Inquisitiveness’ and ‘Accomplishment’. As discussed in the ‘Inquisitiveness’ section, *inquisitiveness* is essential for individuals to engage in learning independently, specifically involving active questioning. Considering this active questioning aspect, the relationship between ‘Inquisitiveness’ and ‘Accomplishment’ is logical, since active questioning would not occur if the learner did not believe in their ability to successfully complete learning tasks or lacked a sense of *accomplishment.*

### Implementation

Once a learner recognises the need for learning, the ‘Implementation’ phase begins, with formulating learning goals, identifying resources, choosing learning strategies, and evaluating the outcomes [7]. While SR, like SRL and SDL, views learning as a cyclical process [23], the steps are not necessarily sequential in every learning process. Each step is informed by other steps and influenced by components such as self-observation, self-judgement, and self-reaction [5]. Learners with high levels of SR are expected to monitor each step in their *implementation*, reflect on outcomes [44], and adapt their strategies accordingly [23].

The items loaded onto the ‘Implementation’ factor in our SR Scale clearly represent these steps, encompassing goal setting (SR 18: ‘I set my own learning objectives’), resource identification (SR 17: ‘To start learning, I organise relevant available learning materials’), adopting/adapting learning strategies (SR19: ‘I plan my learning in advance’), and monitoring (SR30: ‘I evaluate my learning’).

A moderate covariance was observed between ‘Implementation’ and ‘Inquisitiveness’ and between ‘Implementation’ and ‘Accomplishment’ (0.55 and 0.59, respectively; Fig. 1). This relationship, especially when SR is the focus, can be explained by the fact that learners need to be *inquisitive*, driven by a curiosity for learning, and have an adequate sense of *accomplishment* to initiate a learning process and *implement* their learning strategies [35, 43].

### Independence

‘Independence’ explained 2.54% of the variance in our model, involving three items: ‘Ican find resources by myself’ (SR10), ‘I learn independently to other people’ (SR13), and ‘I attempt to independently solve learning challenges’ (SR21). These items represent different aspects of independent learning. According to Moore’s definition [45], *independent learning* occurs when learning takes place in a time and place separate from teaching, with the learner having an influence at least equal to the teacher, in setting goals, identifying resources, and making decisions for evaluation. Particularly, *independence* in learning is governed by distinct dimensions: time, place, pace, content, medium, technology, method, relationship, disclosure, and delegation [46]. It is important to emphasise that *independence,* neither in its definition nor as a factor explaining SR aptitudes, does not equate to learning alone and without any support. Indeed, in modern perspectives, while a learner can be totally independent of a teacher, their *independence* is multifaceted and constantly shifting. That is, they can delegate control in any of the aforementioned dimensions when needed and take it back when it is not [47].

To explain the moderate-to-large covariances observed between ‘Independence’ and all other factors (0.72 with ‘Accomplishment’, 0.66 with ‘Inquisitiveness’, and 0.40 with ‘Implementation’; Fig. 1), we again refer to Moore’s definition [45]. As *independent learning* occurs distantly from teaching, a lack of direct engagement with others might lead to a diminished sense of relatedness [47], which needs to be supported for intrinsic motivation to arise [48]. In this sense, we posit that learners’ innate *inquisitiveness* will, at least partially, fuel intrinsic motivation to mitigate the diminished sense of relatedness; hence, the observed relationship between ‘Independence’ and ‘Inquisitiveness’. Additionally, as the definition of *independent learning* emphasises learner autonomy [45], learners require sufficient confidence in their learning competence and a sense of *accomplishment* to make autonomous choices and take control of their learning [49]. Finally, referring to the definition of SR [23], we expect learners with high SR levels to *independently* initiate their learning and *implement* their learning strategies. Therefore, some degree of relationship between ‘Independence’ and ‘Implementation’ is expected. We emphasise that learners with higher levels of *independence* in learning, and by extension, higher levels of SR, are not necessarily independent in every learning context. Moreover, as Dron argues, ‘[t]here is no such thing as completely independent learning, at least in an educational context, because all learning depends on others, whether now or in the past’ (p. 62) [47].

### Generalisability

Based on the results of the multiple groups invariance analyses, the SR Scale demonstrated consistent factor loadings across gender groups (Δ*χ*^2^ = 24.982, Δ*df* = 16, *p* = 0.070) and courses of study (Δ*χ*^2^ = 21.670, Δ*df* = 16, *p* = 0.154; see the ‘Multiple Groups Invariance Analyses’ section of ‘Results’). This indicates that the scale enables valid comparisons of relationships between latent constructs (i.e. ‘Inquisitiveness’, ‘Accomplishment’, ‘Implementation’, and ‘Independence’) and the final items, unaffected by demographic or field of study—at least within health professions.

Overall, our findings align with the theoretical perspective [9] that adult learners are inherently self-directed—albeit to varying degrees—and, by extension, possess some level of SR aptitudes. Consequently, we believe the SR Scale and the four-factor model proposed in this study may have broad applicability among adult learners.

### Future Directions

It is believed that high levels of SR are essential for learners, particularly health professionals, to thrive as lifelong learners and demonstrate competence in professional practice [23]. As such, greater emphasis should be placed on developing these aptitudes in health professions education. The SR Scale provides a valuable tool for future research, enabling investigation of factors influencing SR aptitudes and informing the development of educational strategies aimed at nurturing and enhancing SR in learners.

### Limitations

A key limitation of this study is its focus on English-speaking countries. Although students from various backgrounds and health disciplines were included, the similarities in the educational systems of these Commonwealth countries may limit broader applicability. This highlights the importance of conducting further validation studies in more diverse educational settings.

This study used the entire sample for both EFA and CFA (*n* = 315). It is acknowledged that some researchers argue that performing CFA on the same sample used for model creation via EFA risks circular reasoning and overfitting [50, 51]. To mitigate this, they advise against conducting CFA as a follow-up analysis to EFA [28] and instead recommend performing cross-validation by creating subsamples, conducting EFA on one subsample, and CFA on the other [52, 53]. Others, however, suggest that a split-sample strategy may be less effective than the whole-sample strategy for evaluating the factor structure and is only viable for large samples [54]. Given the available sample size (*n* = 315), splitting the sample between EFA and CFA could have been counterproductive, as it would increase the likelihood of Type II error due to the insufficient sample size for a split dataset analysis. Additionally, in our study, the multiple groups invariance analysis demonstrated measurement invariance and stability of the factor structure across different populations. The use of bootstrapping for the CFA provided additional evidence supporting the stability of the factor structure [33].

Finally, while the total sample size was sufficient to establish validity and generalisability, there was a disproportionate number of female respondents. However, measurement invariance was established across genders, indicating that the imbalance did not affect the validity of the findings. Nonetheless, larger samples across different subgroups, particularly students from various health fields or academic levels, may further enhance the applicability of the findings.

## Conclusion

Our study has revealed that the proposed SR Scale is a reliable tool with good content and construct validity for assessing SR in health profession students. According to our analyses, SR aptitudes are primarily influenced by ‘Inquisitiveness’, ‘Accomplishment’, ‘Implementation’, and ‘Independence’. This four-factor model of SR offers a comprehensive perspective on students’overall capacity to take control of their learning as lifelong learners when they identify gaps in their professional knowledge. This understanding will help uncover enablers and barries to SR aptitudes and guide measures to further promote these aptitudes in health professions students and early-career health professionals.

## Supplementary Information

Below is the link to the electronic supplementary material.

Supplementary file 1 (DOCX 50.6 KB)

## Data Availability

The datasets generated during and/or analysed during the current study are available from the corresponding author on reasonable request.
