# Supplementary material for: Development and Validation of a Tool for Evaluating Self-regulated and Self-directed Aptitudes of Learning (SELF-ReDiAL)
Source: Med Sci Educ. 2025 Jul 4;35(5):2429–39. doi: 10.1007/s40670-025-02454-0 (PMC12812129; doi:10.1007/s40670-025-02454-0)
Supplement: Supplementary file 1 — (DOCX 50.6 KB) [file 40670_2025_2454_MOESM1_ESM.docx]

*Medical Science Educator*

**Online Resource 1**

**Development and validation of a tool for evaluating self-regulated and self-directed aptitudes of learning (SELF-ReDiAL)**

Arash Arianpoor^1,2^, Silas C.R. Taylor^1^, Cherie Lucas^3^, Craig S. Webster^4,5^, Marcus Henning^4^, Ernesta Sofija^6^, Matthew J. Boyd ^7^, Theresa Charrois^8^, Jamie Kellar ^9^, Jason Perepelkin^10^, Lorraine Smith^11^, Revathy Mani^12^, Efi Mantzourani^13^, Catherin Marley^14^, Boaz Shulruf^1,4^, Pin-Hsiang Huang^1,15*^

1. Office of Medical Education, Faculty of Medicine and Health, The University of New South Wales, Sydney, NSW, Australia
2. School of Biomedical Sciences, Faculty of Medicine and Health, The University of New South Wales, Sydney, NSW, Australia
3. School of Population Health, Faculty of Medicine and Health, The University of New South Wales, Sydney, NSW, Australia
4. Centre for Medical and Health Sciences Education, School of Medicine, University of Auckland, Auckland, New Zealand
5. Department of Anaesthesiology, Faculty of Medical and Health Sciences, University of Auckland, Auckland, New Zealand
6. School of Medicine and Dentistry, Griffith University, Gold Coast, QLD, Australia
7. Division of Pharmacy Practice and Policy, School of Pharmacy, University of Nottingham, Nottingham, United Kingdom
8. Faculty of Pharmaceutical Sciences, The University of British Columbia, Vancouver British Columbia, Canada
9. Leslie Dan Faculty of Pharmacy, University of Toronto, Toronto, Canada
10. College of Pharmacy & Nutrition, University of Saskatchewan, Saskatoon, Saskatchewan, Canada
11. Sydney Pharmacy School, Faculty of Medicine and Health, The University of Sydney, NSW, Australia
12. School of Optometry and Vision Science, Faculty of Medicine and Health, The University of New South Wales, Sydney, NSW, Australia
13. School of Pharmacy and Pharmaceutical Sciences, Cardiff University, Cardiff, Wales, United Kingdom
14. Faculty of Medicine and Health, The University of New South Wales, Sydney, NSW, Australia
15. Department of Medical Humanities and Medical Education, College of Medicine, National Yang Ming Chiao Tung University, Taipei, Taiwan

*Corresponding author

Pin-Hsiang Huang, PhD

Academic Development Lead

Office of Medical Education, Faculty of Medicine and Health, The University of New South Wales, Sydney, NSW, Australia

Email: [pin-hsiang.huang@unsw.edu.au](mailto:pin-hsiang.huang@unsw.edu.au)

**Table S1. Initial list of items**

| Code | Item | Code | Item |
| --- | --- | --- | --- |
| SR1 | I search for possibilities to learn new things. | SR16 | To start learning, I identify relevant available resources. |
| SR2 | I learn to get top grades. | SR17 | To start learning, I organise relevant available learning materials. |
| SR3 | I learn to improve myself. | SR18 | I set my own learning objectives. |
| SR4 | My reason for learning is to gain personal benefit. | SR19 | I plan my learning in advance. |
| SR5 | I learn to satisfy my curiosity. | SR20 | I learn through explaining to others. |
| SR6 | I enjoy learning new things. | SR21 | I attempt to independently solve learning challenges. |
| SR7 | I seek to learn beyond the stated requirement. | SR22 | I appraise the utility of learning resources. |
| SR8 | I can learn anything relevant to my needs. | SR23 | I deliberately integrate new knowledge with my existing knowledge. |
| SR9 | I am aware of my learning capabilities. | SR24 | I adapt my learning strategies to meet challenges. |
| SR10 | I can find resources by myself. | SR25 | I complete my learning despite challenges. |
| SR11 | I take responsibility for my learning. | SR26 | I meet my learning objectives. |
| SR12 | I can learn anything relevant to my needs in my field of study. | SR27 | I meet my learning needs on schedule. |
| SR13 | I learn independently to other people. | SR28 | I meet my learning needs fully. |
| SR14 | I welcome challenges in learning. | SR29 | I seek out assurance of the quality of my learning. |
| SR15 | I can master new skills. | SR30 | I evaluate my learning. |

**Table S2: Frequency of recorded responses by university and course of study**

|  | | University | | | | | | | | | | | |  |
| --- | --- | --- | --- | --- | --- | --- | --- | --- | --- | --- | --- | --- | --- | --- |
|  |  | Griffith University | University of Sydney | University of Alberta | University of Auckland | University of British Columbia | University of East Anglia | University of Nottingham | University of Saskatchewan | University of Tasmania | University of Toronto | University of New South Wales | Joint Medical Program at University of Newcastle and University of New England | Total |
| **Course of study** | Bachelor of Biomedical Sciences | 3 (11.1%) | None | None | None | None | None | None | None | None | None | None | None | 3 (1.0%) |
|  | Bachelor of Exercise Physiology | None | None | None | None | None | None | None | None | None | None | 7 (9.6%) | None | 7 (2.2%) |
|  | Bachelor of Exercise Science | 2 (7.4%) | None | None | None | None | None | None | None | None | None | 3 (4.1%) | None | 5 (1.6%) |
|  | Bachelor of Health Sciences | 1 (3.7%) | None | None | 25 (30.1%) | None | None | None | None | None | None | None | None | 26 (8.3%) |
|  | Bachelor of Medical Imaging | None | None | None | 3 (3.6%) | None | None | None | None | None | None | None | None | 3 (1.0%) |
|  | Bachelor of Medical Laboratory Science | 2 (7.4%) | None | None | None | None | None | None | None | None | None | None | None | 2 (0.6%) |
|  | Bachelor of Nutrition/Dietetics | 8 (29.6%) | None | None | None | None | None | None | None | None | None | 1 (1.4%) | None | 9 (2.9%) |
|  | Bachelor of Occupational Therapy (Honours) | 2 (7.4%) | None | None | None | None | None | None | None | None | None | None | None | 2 (0.6%) |
|  | Bachelor of Pharmacology and Toxicology | 1 (3.7%) | None | None | None | None | None | None | None | None | None | None | None | 1 (0.3%) |
|  | Bachelor of Pharmacy | None | 11 (78.6%) | None | None | None | None | None | None | None | None | 3 (4.1%) | None | 14 (4.4%) |
|  | Bachelor of Psychology | 2 (7.4%) | None | None | None | None | None | None | None | None | None | None | None | 2 (0.6%) |
|  | Bachelor of Vision Science/Optometry | None | None | None | None | None | None | None | None | None | None | 21 (28.8%) | None | 21 (6.7%) |
|  | Doctor of Pharmacy | None | None | 17 (100.0%) | None | 4 (100.0%) | None | None | 23 (100.0%) | None | 17 (100.0%) | None | None | 61 (19.4%) |
|  | Master of Audiology | None | None | None | 2 (2.4%) | None | None | None | None | None | None | None | None | 2 (0.6%) |
|  | Master of Health Leadership | None | None | None | 6 (7.2%) | None | None | None | None | None | None | None | None | 6 (1.9%) |
|  | Master of Health Sciences | None | None | None | 6 (7.2%) | None | None | None | None | None | None | None | None | 6 (1.9%) |
|  | Master of Nutrition/Dietetics | None | None | None | 1 (1.2%) | None | None | None | None | None | None | None | None | 1 (0.3%) |
|  | Master of Pharmacy | 1 (3.7%) | 3 (21.4%) | None | None | None | 1 (100.0%) | 12 (48.0%) | None | None | None | None | None | 17 (5.4%) |
|  | Master of Public Health | 1 (3.7%) | None | None | 3 (3.6%) | None | None | None | None | None | None | None | None | 4 (1.3%) |
|  | Medicine | 4 (14.8%) | None | None | 22 (26.5%) | None | None | 13 (52.0%) | None | 22 (100.0%) | None | 38 (52.1%) | 9 (100.0%) | 108 (34.3%) |
|  | PG Certificate in Clinical Education | None | None | None | 2 (2.4%) | None | None | None | None | None | None | None | None | 2 (0.6%) |
|  | PG Diploma in Health Sciences | None | None | None | 11 (13.3%) | None | None | None | None | None | None | None | None | 11 (3.5%) |
|  | PG Diploma in Palliative Care | None | None | None | 1 (1.2%) | None | None | None | None | None | None | None | None | 1 (0.3%) |
|  | PG Diploma in Public Health | None | None | None | 1 (1.2%) | None | None | None | None | None | None | None | None | 1 (0.3%) |
|  | Total | 27 (100.0%) | 14 (100.0%) | 17 (100.0%) | 83 (100.0%) | 4 (100.0%) | 1 (100.0%) | 25 (100.0%) | 23 (100.0%) | 22 (100.0%) | 17 (100.0%) | 73 (100.0%) | 9 (100.0%) | 315 (100.0%) |

**Table S3. Final list of items for evaluation of self-regulated and self-directed aptitudes of learning (SELF-ReDiAL)**

| Latent construct | Item | Responses options | | | | | |
| --- | --- | --- | --- | --- | --- | --- | --- |
|  |  | Never | Rarely | Occasionally | Often | Very Often | Always |
| Inquisitiveness | I learn to satisfy my curiosity |  |  |  |  |  |  |
|  | I search for possibilities to learn new things |  |  |  |  |  |  |
|  | I enjoy learning new things |  |  |  |  |  |  |
|  | I seek to learn beyond the stated requirement |  |  |  |  |  |  |
|  | I deliberately integrate new knowledge with my existing knowledge |  |  |  |  |  |  |
|  | I learn to improve myself |  |  |  |  |  |  |
|  | I welcome challenges in learning |  |  |  |  |  |  |
|  | My reason for learning is to gain personal benefit |  |  |  |  |  |  |
| Accomplishment | I meet my learning needs fully |  |  |  |  |  |  |
|  | I meet my learning needs on schedule. |  |  |  |  |  |  |
|  | I meet my learning objectives. |  |  |  |  |  |  |
|  | I am aware of my learning capabilities. |  |  |  |  |  |  |
|  | I complete my learning despite challenges. |  |  |  |  |  |  |
| Implementation | I evaluate my learning. |  |  |  |  |  |  |
|  | I set my own learning objectives. |  |  |  |  |  |  |
|  | I plan my learning in advance |  |  |  |  |  |  |
|  | To start learning, I organise relevant available learning materials. |  |  |  |  |  |  |
| Independence | I learn independently to other people. |  |  |  |  |  |  |
|  | I attempt to independently solve learning challenges. |  |  |  |  |  |  |
|  | I can find resources by myself. |  |  |  |  |  |  |

For scoring purposes, "Never" is assigned a score of 1, and "Always" a score of 6.

**Table S4. Confirmatory factor analysis path estimates: Standardised regression weights with bootstrap standard errors and confidence intervals**

| **Parameter** | **Estimate^a^** | **95% confidence interval^b^** | | **P^b^** | **SE^c^** | **SE-SE^d^** | **Mean** | **Bias^e^** | **SE-Bias^f^** |
| --- | --- | --- | --- | --- | --- | --- | --- | --- | --- |
|  |  | **Lower** | **Upper** |  |  |  |  |  |  |
| SR14_Score ← Inquisitiveness | 0.708 | 0.628 | 0.762 | 0.005 | 0.033 | 0.001 | 0.709 | 0.001 | 0.001 |
| SR3_Score ← Inquisitiveness | 0.756 | 0.693 | 0.81 | 0.003 | 0.029 | 0.001 | 0.757 | 0.001 | 0.001 |
| SR23_Score ← Inquisitiveness | 0.633 | 0.555 | 0.701 | 0.002 | 0.037 | 0.001 | 0.632 | -0.001 | 0.001 |
| SR7_Score ← Inquisitiveness | 0.665 | 0.589 | 0.732 | 0.002 | 0.036 | 0.001 | 0.665 | 0 | 0.001 |
| SR6_Score ← Inquisitiveness | 0.705 | 0.638 | 0.766 | 0.002 | 0.033 | 0.001 | 0.704 | 0 | 0.001 |
| SR1_Score ← Inquisitiveness | 0.669 | 0.595 | 0.743 | 0.002 | 0.038 | 0.001 | 0.669 | 0 | 0.001 |
| SR4_Score ← Inquisitiveness | 0.438 | 0.336 | 0.528 | 0.003 | 0.049 | 0.001 | 0.439 | 0.001 | 0.002 |
| SR5_Score ← Inquisitiveness | 0.701 | 0.622 | 0.759 | 0.004 | 0.034 | 0.001 | 0.703 | 0.001 | 0.001 |
| SR9_Score ← Accomplishment | 0.63 | 0.549 | 0.702 | 0.002 | 0.039 | 0.001 | 0.628 | -0.002 | 0.001 |
| SR26_Score ← Accomplishment | 0.785 | 0.717 | 0.836 | 0.002 | 0.028 | 0.001 | 0.785 | -0.001 | 0.001 |
| SR27_Score ← Accomplishment | 0.675 | 0.594 | 0.74 | 0.003 | 0.037 | 0.001 | 0.675 | 0 | 0.001 |
| SR28_Score ← Accomplishment | 0.798 | 0.735 | 0.849 | 0.003 | 0.029 | 0.001 | 0.799 | 0.001 | 0.001 |
| SR25_Score ← Accomplishment | 0.691 | 0.619 | 0.754 | 0.002 | 0.034 | 0.001 | 0.69 | -0.001 | 0.001 |
| SR30_Score ← Implementation | 0.682 | 0.554 | 0.78 | 0.003 | 0.057 | 0.001 | 0.682 | 0.001 | 0.002 |
| SR18_Score ← Implementation | 0.584 | 0.45 | 0.683 | 0.003 | 0.057 | 0.001 | 0.585 | 0 | 0.002 |
| SR17_Score ← Implementation | 0.484 | 0.359 | 0.589 | 0.003 | 0.057 | 0.001 | 0.482 | -0.001 | 0.002 |
| SR19_Score ← Implementation | 0.661 | 0.54 | 0.764 | 0.002 | 0.057 | 0.001 | 0.659 | -0.002 | 0.002 |
| SR21_Score ← Independence | 0.655 | 0.561 | 0.746 | 0.002 | 0.046 | 0.001 | 0.654 | -0.001 | 0.001 |
| SR10_Score ← Independence | 0.675 | 0.578 | 0.754 | 0.003 | 0.043 | 0.001 | 0.676 | 0.001 | 0.001 |
| SR13_Score ← Independence | 0.604 | 0.501 | 0.691 | 0.003 | 0.048 | 0.001 | 0.605 | 0.001 | 0.002 |

^a^Standardised regression weights

^b^Bias-corrected percentile method

^c^SE represents the bootstrap *standard error*.

^d^SE-SE represents the standard error of the bootstrapped standard error estimate.

^e^Bias refers to the difference between the average of the bootstrap estimates and the original sample estimate.

^f^SE-Bias represents the standard error of the bias estimate.
